# Supplementary material for: Causal Relationship between Aging and Anorexia Nervosa: A White-Matter-Microstructure-Mediated Mendelian Randomization Analysis
Source: Biomedicines. 2024 Aug 16;12(8):1874. doi: 10.3390/biomedicines12081874 (PMC11351342; doi:10.3390/biomedicines12081874)
Supplement: Supplementary file 1 [file biomedicines-12-01874-s001.zip › Supplementary Figure/Figure S8/Leaveoneout plot of MR analysis for TL to ubm-b-1607.pdf]

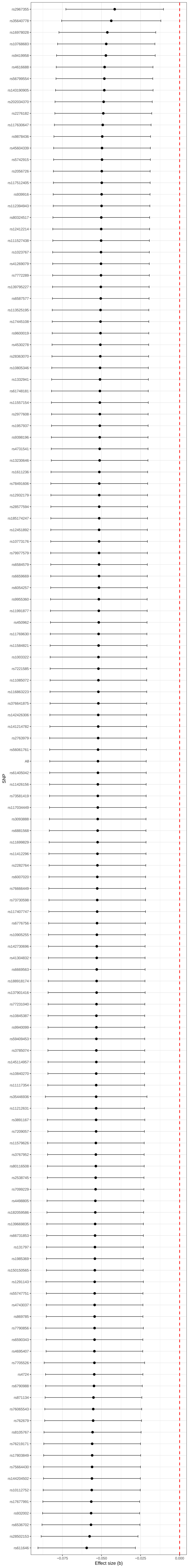

rs2967355
rs35640778
rs16978028
rs10768683
rs9419958
rs4616688
rs56799554
rs143190905
rs202034370
rs2276182
rs117630647
rs9878436
rs45604339
rs5742915
rs2056726
rs117512405
rs939916
rs112394943
rs80324517
rs12412214
rs111527438
rs1023767
rs41269079
rs7772289
rs139795227
rs6587577
rs113525195
rs17445108
rs9600019
rs4530278
rs28363070
rs10805346
rs1332941
rs61748181
rs11557154
rs2977608
rs1957937
rs9398196
rs4731541
rs13230646
rs1611236
rs78491606
rs12932179
rs28577594
rs185174247
rs12451892
rs10773176
rs79977579
rs6584579
rs6659669
rs6054257
rs9955360
rs11991877
rs450962
rs11769630
rs11584821
rs1003322
rs7221585
rs11085072
rs116863223
rs376641875
rs142426306
rs141214782
rs2763979
rs56061761
All
rs61405042
rs11426156
rs73581419
rs117034449
rs3093888
rs6881568
rs11699829
rs11412296
rs2282764
rs6007020
rs76666449
rs73730598
rs117407747
rs6776756
rs10905255
rs142730696
rs41304832
rs6669563
rs188918174
rs137901416
rs77231040
rs10845387
rs9940099
rs59409453
rs3785074
rs145114957
rs10840270
rs11117354
rs35446936
rs11212631
rs3891167
rs7209057
rs11579626
rs3767952
rs80116508
rs2538745
rs7099229
rs4498805
rs182059586
rs139669835
rs66731853
rs131797
rs1985369
rs150150565
rs1291143
rs55747751
rs4743037
rs869785
rs7790856
rs6590343
rs4695407
rs7705526
rs4724
rs6790988
rs871134
rs76065543
rs762679
rs8105767
rs76219171
rs17803849
rs75664430
rs144204502
rs10112752
rs17677991
rs932002
rs6536702
rs28502153
rs611646
SNP
−0.075
−0.050
−0.025
0.000
Effect size (b)
